# Luminance gradient configuration determines perceived lightness in a simple geometric illusion

**DOI:** 10.3389/fnhum.2014.00977

**Published:** 2014-12-05

**Authors:** Maria Pereverzeva, Scott O. Murray

**Affiliations:** Department of Psychology, University of WashingtonSeattle, WA, USA

**Keywords:** lightness, luminance gradient, illusion, brightness

## Abstract

Accurate perception of surface reflectance poses a significant computational problem for the visual system. The amount of light reflected by a surface is affected by a combination of factors including the surface’s reflectance properties and illumination conditions. The latter are not limited by the strength of the illuminant but also include the relative placement of the light illuminating the surface, the orientation of the surface and its 3d shape, all of which result in a pattern of luminance gradients across the surface. In this study we explore how luminance gradients contribute to lightness perception. We introduce a novel, simple lightness illusion. It consists of six separate checks, organized in rows of two. Each check has a negative luminance gradient across it. The top and the bottom rows are the same: with the darker check on the left, and the lighter check on the right. Two checks in the middle row are identical; however, the check on the right appears darker than the check on the left. As there are no shared borders between the checks, simultaneous contrast cannot explain the effect. However, there are multiple possible explanations including spatial filtering (Blakeslee and McCourt, 2004) or some higher-order mechanism such as perceptual grouping or amodal completion. Here, we explore these possibilities by manipulating the luminance configurations and the gradient slopes of the checks.

## Introduction

**Figure 1:**
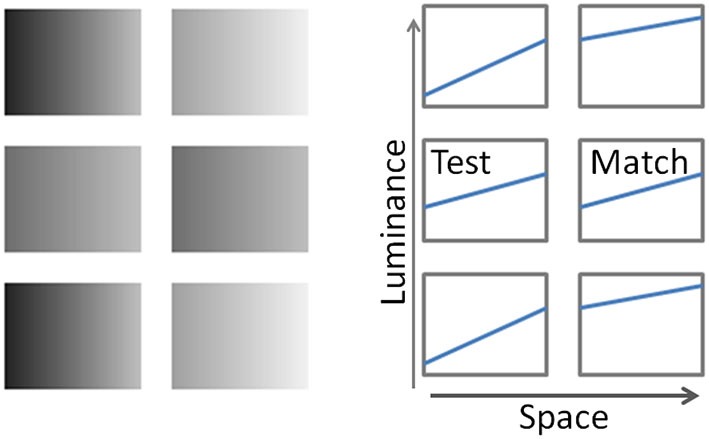
**Left panel: The Gradient illusion**. The two checks in the middle row are identical; however, the check on the right appears darker than the one on the left. Right panel: schematic depiction of luminance profiles of corresponding “checks”.

Accurate perception of surface reflectance poses a significant computational problem for the visual system. The amount of light reflected by a surface is affected by a combination of factors including the surface’s reflectance properties and illumination conditions. The latter are not limited by the strength of illuminant but also include the relative placement of the light illuminating the surface, the orientation of the surface and its 3d shape, all of which result in a pattern of luminance gradients across the surface. In this study we explore how presence of luminance gradients in parts of the image contributes to lightness perception. Here, we introduce a novel illusion (Figure 1). It consists of six checks, organized in rows of two. Each check has a linear luminance gradient across it; for example, in Figure 1, the luminance increases from the left to the right side of each check (the luminance profiles of each check are shown schematically in the right panel of Figure 1). The top and the bottom rows are the same: with the overall darker check on the left, and the overall lighter check on the right. The two checks in the middle row are identical; however, the check on the right appears darker than the check on the left.

This illusion can potentially be considered a simplification of Adelson’s (2000) checker shadow illusion, and a modification of Kitaoka’s (2005) simplified rendering of it, “Adelson’s checker-shadow illusion-like gradation lightness illusion”, with one critical difference: the gradient checks in our display are separated, while Adelson’s and Kitaoka’s displays use continuous checkerboards in which at least part of the observed effect can be explained by border contrast, a change in surface lightness due to different luminances of the immediate surrounds (Cornsweet, 1970; Kingdom and Moulden, 1988; Hung et al., 2007). Because there are no shared borders between the checks in our illusion, simple edge integration models (Land and McCann, 1971; Hurlbert and Poggio, 1988; Rudd and Zemach, 2004; Rudd and Popa, 2007, for review see Gilchrist et al., 1999) cannot explain the effect.

There are, however, multiple potential explanations for our illusion including spatial filtering (Blakeslee and McCourt, 1999, 2004) and higher-order mechanisms such as amodal completion—when objects are perceived as whole even though only parts of them are visible. For example, simultaneous contrast effects by amodal completion were observed in several studies, including Bressan’s Dungeon illusion (Bressan, 2001, 2006a,b) and Boyaci et al’s modified Craik–O’Brien-Cornsweet illusion (Boyaci et al., 2010). In addition, illumination discounting models might predict that the change in lightness is related to the overall luminance distribution in the scene, and a 3d interpretation of the surfaces (Bergström, 1977, 1982; Bergström et al., 1984; Knill and Kersten, 1991; Arend, 1994; Bloj et al., 1999). To explore these possibilities, and better understand the observed effect, we manipulated the luminance configurations and gradient signs of the checks.

## Methods

### Subjects

Seven subjects (five of whom were naïve to the purpose of the experiment), aged 23–44 participated in the experiments. All had normal or corrected to normal visual acuity. Prior to testing, in accordance with the University of Washington Human Subjects Institutional Review Board, an informed written consent was obtained from the subjects. All seven subjects participated in conditions of experiment 1 and 3 with Configurations 1, 2 and 7. Five of these subjects participated in the experiment 2 with Configuration 5, and three subjects—in experiments 2 and 3 with Configurations 3, 4, and 6.

### Apparatus and stimulus specifications

The apparatus consisted of a ViewSonic G90fB on an ATI Radeon HD 4800 series color graphics display monitor controlled by a Dell Studio XPS 435T PC, and calibrated with a PR 650 spectroradiometer (Photo Research, CA). The monitor had a peak luminance of 150 cd/m^2^ and a black level of 0.16 cd/m^2^. It extended 40 by 29° of visual angle at a viewing distance of 57.0 cm. The test stimuli were six 3.5 by 2.7° achromatic rectangular “checks” embedded in a full-field white surround, and positioned centrally. The checks were organized into three rows of two checks each separated by 0.8° gaps vertically and horizontally.

For simplicity, all stimulus luminances are specified in Instrument Luminance (IL), defined as 100%*(L-Lmin)/(Lmax-Lmin), where L is the stimulus luminance, Lmin is the black level of the monitor and Lmax is the maximal available luminance of the display. When present, all linear luminance gradients will be defined as (%IL(right) − %IL(left))/size(deg), where %IL(right) and %IL(left) are respective ILs on the left-hand and the right-hand sides of the gradient and size is the width of the rectangle), in degrees of visual angle. The stimulus luminance profiles are adequately represented by two values: space-average luminance and linear gradient.

An example of luminance profile depiction is shown in the right panel of Figure 1. Each rectangle represents the respective “check” of the illusion; with luminance profile as a function of spatial location on each check, shown separately.

**Figure 2:**
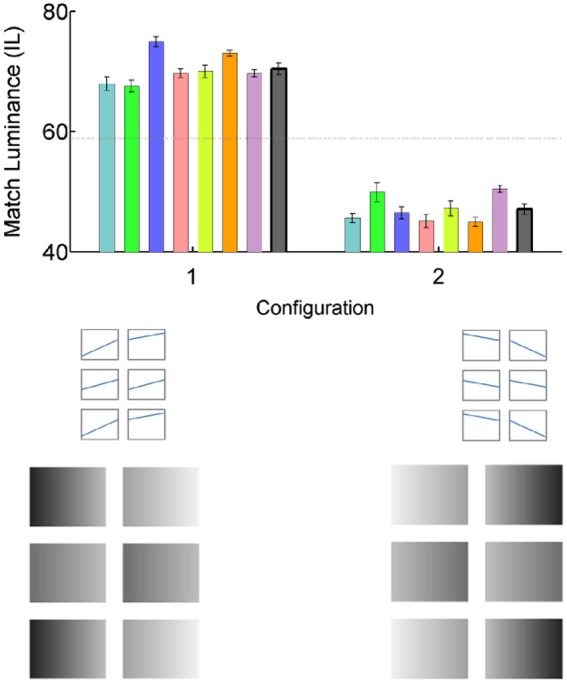
**The gradient illusion**. The graph shows luminance match settings, when the overall luminance of the Match check (mid-row, right) was adjusted to match the Test check (mid-row, left), for individual subjects (error bars are SE for 10 matches done by each subject)—colored bars; and the mean match of 7 subjects error bars are the SE of the mean)—gray bars. Veridical match is indicated by a horizontal dotted line through 58.8 IL. Luminance profiles of each check by location are shown schematically for two configurations in the middle set of panels, plotting luminance as a function of location on each check. The lower set of panels show the gradient configurations (scaled), as presented to subjects. In Configuration 1, the Match check appears darker than the Test. In Configuration 2 this effect is reversed.

In all configurations of this study, the stimuli were six checks organized into three rows of two checks each. In each configuration, the originally presented checks in the middle row (Test and Match) were always physically identical to each other, and had the mean IL of 58.8. When the gradient was present, the Test and Match gradient was either 9.0 or −9.0 (as in Configuration 2 of Figure 2). In each configuration, the top and the bottom “flanker’ rows were always identical, and with an exception of Configuration 5, which consisted of lighter and darker checks, with respective mean luminances of 78.6 and 44.1 IL. When gradients were present in the flanker checks, they were 9.1 and 17.4, respectively (or −9.1 and −17.4). In Configuration 5 the luminances of all flanker checks were 58.8 IL, same as the overall luminance of the Test and Match checks. The surround luminance was 100 IL throughout the experiment.

### Procedure

The subjects were instructed to adjust the luminance of Match checks to match the appearance of the Test checks. The adjustment was done in luminance steps of +/−1 IL, by pressing “1” or “2” keys. Only the overall luminance of the Match was changed by the adjustments; the gradient was held constant. Once the subjects were satisfied with their settings, the matches were recorded by pressing a “7” key, which also initiated a new trial. The judgments were made under free viewing conditions, with unlimited viewing time; each trial was preceded by a 20 s adaptation period. A total of 10 trials per Configuration were collected. Each experimental session consisted of a practice run followed by pseudo-randomly chosen set of configurations, and lasted about an hour.

## Results

### Condition 1: the illusion

The results of the first experiment characterize the magnitude of the original illusion. To control for possible side bias, the illusion was presented in two different gradient orientations (Figure 2: stimulus Configurations 1 and 2: the second configuration is the mirror image of the first). The colored bars in Figure 2 show individual luminance settings of the match rectangle, required to match the appearance of the test rectangle (veridical match is 58.8 IL, shown by the horizontal dashed line). The gray bars are mean matches of seven subjects, with error bars representing SE of the mean. The data indicate that for all subjects, the match rectangle in Configuration 1 appeared much darker than the test (and had to be adjusted to the mean luminance of 70.4 +/− 1.01 IL to match the appearance of the test), while in Configuration 2 it appeared much lighter than the test (and had to be adjusted to the mean luminance of 47.1 +/− 0.85 IL to match the appearance of the test).

### Condition 2: effect of the spatially uniform flankers

**Figure 3:**
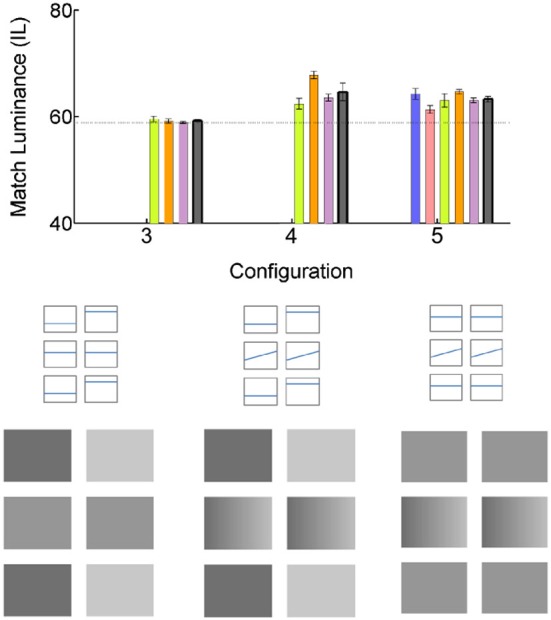
**Effect of the uniform luminance flankers**. All conventions are as in Figure 2. The bars of the same color plot the data of the same subjects as in Figure 2.

To test the possibility that the effect could have nothing to do with the gradients we removed gradients from the original illusion shown in Figure 1. The new configuration consisted of two identical gray checks in the middle row, surrounded by an upper and lower flanker rows, in which the left checks were darker than the right. In case of amodally completed contrast, the Match check should appear darker than the Test. However, the data plotted in Figure 3 (Configuration 3), indicate that was not the case: there were no deviations from the veridical luminance match at 59.1830 +/− 0.2008 IL.

We continued to explore this question by restoring the gradients to the middle checks (Configuration 4). Surprisingly, it resulted in illusory brightness change: The Match check appeared to be darker than the Test, resulting in the luminance match setting of 64.5915 +/− 1.6369 IL. The results were especially surprising given that the only change from Configuration 3, in which no illusory brightness difference was observed, was an addition of gradient to the middle row checks.

Even more surprisingly, replacing the flankers with identical uniform gray checks, did not change the illusion: the gradient Match check still appeared darker than the Test, with the resulting luminance match setting of 63.2647 +/− 0.5834 IL (Configuration 5). The effect is, however, noticeably smaller than that in the main illusion (Configuration 1).

### Condition 3: effect of the gradient flankers. Remote gradients in flankers affect lightness perception in uniform checks

**Figure 4:**
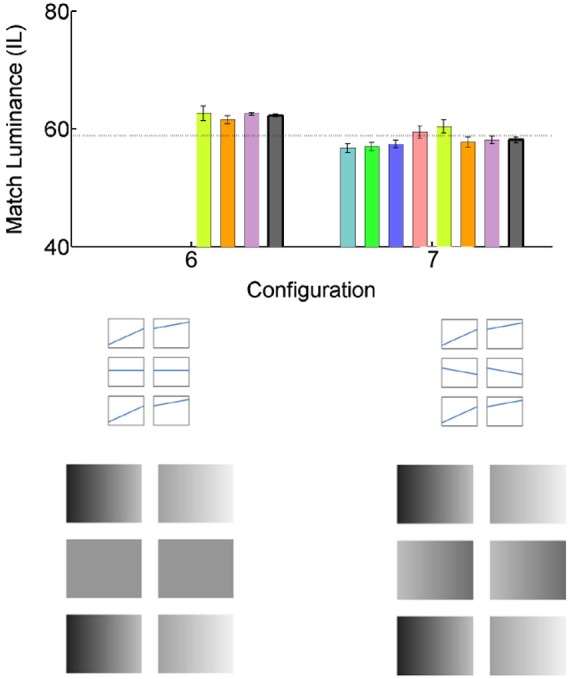
**Effect of the gradient luminance flankers**. All conventions are as in Figure 2.

We have shown that the lightness of the middle checks is not affected by the overall luminance of the flankers (Configurations 3, 4 and 5, above), but is it affected by luminance gradients in the flankers? To explore this possibility, we tested Configuration 6 (Figure 4): Luminance gradients on the flankers and flat luminance profiles of the middle checks. We found that introducing positive gradients to the flankers results in the darker appearance of the Match check, with the resulting luminance match setting of 62.2549 +/− 0.2254 IL. Finally, we investigated the interaction between the flanker gradients and those in the middle checks (Configuration 7). Adding negative gradients to the middle checks decreases (and for some subjects, reverses) the illusory darkness in the Match check with the resulting luminance match setting of 58.1353 +/− 0.5037 IL.

## Discussion

We have demonstrated a novel illusory lightness induction effect from remote luminance gradients. The effect is strikingly large; about 12% IL, which translates into about 20% luminance increase (or decrease) in match settings, as compared to veridical. While we can dismiss the possibility that this effect is due to border contrast—since there is no border contact between the checks—other possibilities need to be considered. One of such possibilities is amodal completion. The idea is that the visual system may interpret our illusory setup as two sets of surfaces: the one in front consisting of white crossbars, partially blocking from view the surface behind it. This back surface consists of contiguous checks similar to those in Adelson’s (2000) checker shadow illusion, and Kitaoka’s simplified interpretation of it (“Adelson’s checker-shadow illusion-like gradation lightness illusion”). The illusory lightness induction in this amodally completed surface may be responsible for the observed effect. If this were the case, we should expect the similar effect in the similar setups, particularly in the Configuration 3; however, no contrast effect was observed.

Spatial filtering could be another possible explanation. Spatial luminance configurations of the visual displays, both adjacent to and remote from the test region have been shown to affect a region’s lightness (Blakeslee and McCourt, 2005). Spatial filtering models (Blakeslee and McCourt, 1999, 2004) can account for many such effects. Indeed, the data from the stimulus configurations 1, 4, 3, 6, and 7 show a monotonic relationship with quantitative predictions of the ODOG model (McCourt and Blakeslee, personal communication), suggesting that the ODOG model can account for some of our observed effects. However, the ODOG model also predicts a relatively large illusory induction effect in Configuration 3 as well as in Configuration 7, which were not observed in our data. In addition, the ODOG model predicts a much larger induction effect in Configuration 4 than in Configuration 5, due to the large difference in luminance. However, the induction effects in Configurations 4 and 5 were about the same, indicating that spatial filtering alone cannot account for all of the observed effects.

By comparing configurations with and without the gradients, we can conclude that the gradient strongly contributed to the lightness induction effect. For instance, as seen in Configuration 5, as well as in comparison between the Configurations 3 and 4, introducing gradients into the Test and Match checks resulted in lightness induction.

Earlier work on illumination estimation and discounting provides an insight into observed gradient effects (Bergström, 1977, 1982; Bergström et al., 1984; Arend and Goldstein, 1987). In the real world of objects and illuminants, the luminance pattern in Configuration 1 would be similar to the luminance pattern produced by a light/dark checkerboard cylinder illuminated from the right and viewed behind a white grating. In this case, the middle-row of checks represent unevenly illuminated “veridically” light and dark checks on the cylinder surface, and the illusion would serve to provide the probable surface interpretation by “discounting” the gradient across all 6 checks. Consistent with this possibility, Knill and Kersten (1991) and Bloj et al. (1999) showed how 3D shape interpretation can radically change surface lightness (or color and/or lightness). Of particular interest is also the work of Bergström (Bergström, 1977, 1982; Bergström et al., 1984), who proposed a model of perceptual analysis, decomposing a stimulus into common and relative components. A common component (here, the gradient) will be associated with the illumination and the relative components (here, the luminances of the checks)—with surface reflectance. Bergström’s model would indicate that the illusion in Configurations 1 and 2 represents a gradient of illumination (light from the right and the left, respectively) on an object. Configuration 7 presents a particularly interesting problem with respect to this model, as it has gradients with opposing directions, which would imply a more complicated decomposition, and a potential scene interpretation as either containing two illuminants, or a combination of concave/convex surfaces. While our data doesn’t have the power of separating between these possibilities, there are earlier studies addressing the question of the gradient inconsistencies (Arend and Goldstein, 1987), including cases where gradient inconsistencies result in gross luminance overestimation (glare phenomena and self-luminosity e.g., Zavagno and Caputo, 2001, 2005; Keil, 2006).

Interestingly, under the conditions tested, the spatial location of the gradient checks did not determine the lightness induction effect (but see McCourt et al., 2013). We tested two different spatial locations: gradients presented concurrently, in the same row (in the Test and Match checks, Configuration 5), and in parallel, in different rows (in the flankers, Configuration 6). Either of these gradient configuration resulted in lightness induction: the Match check was perceived as darker than the Test.

Can we estimate the total effect from an additive combination of different gradient configurations? To answer this question, we can look at the following comparisons: Configuration 3 vs. Configuration 4, and/or Configuration 5: Adding the gradient only to the Test and Match checks results in about 5% IL lightness induction; Configuration 6: adding gradient only to the flankers results in about 4% IL lightness induction, Configuration 1: same-sign gradients in both flankers the test and match result in about 12% IL lightness induction; and finally, Configuration 6 vs. Configuration 7: adding the opposite sign gradient in the Test and Match checks eliminates the flanker effect. In summary, gradient effects in the flankers and in the Test and Match checks interact in a roughly additive fashion: (Configuration 1 effect ≈ Configuration 6 effect + Configuration 5 effect; Configuration 7 effect ≈ Configuration 6 effect − Configuration 5 effect).

In summary, we have presented a novel lightness illusion, which appears to be driven mainly by presence and relative spatial configurations of luminance gradients, interacting in a roughly additive fashion. We were not able to account for the observed effect by either border contrast, amodal completion, or spatial filtering. Further investigation is needed to identify the mechanism(s) causing this illusion.

## Conflict of interest statement

The authors declare that the research was conducted in the absence of any commercial or financial relationships that could be construed as a potential conflict of interest.
